# Artificial Intelligence Reporting Guidelines in Radiology: A Systematic Review

**DOI:** 10.3390/diagnostics16183054

**Published:** 2026-09-20

**Authors:** Katarzyna Ochman, Miłosz Korbaś, Dominika Kaczyńska, Adam Mitręga, Radosław Zaucha, Jakub Kufel

**Affiliations:** 1 Students’ Scientific Association of Computer Analysis and Artificial Intelligence at the Department of Radiology and Nuclear Medicine, Medical University of Silesia, 40-752 Katowice, Poland; 2 Department of Biophysics, Faculty of Medical Sciences in Zabrze, Medical University of Silesia in Katowice, Jordana 18, 40-043 Zabrze, Poland; 3 Department of Radiology and Nuclear Medicine, Medical University of Silesia, 40-752 Katowice, Poland

**Keywords:** artificial intelligence, radiology, diagnostic imaging, medical imaging, reporting guidelines, machine learning

## Abstract

**Background**: Artificial intelligence (AI) is increasingly being used in radiology, prompting the development of reporting guidelines, checklists, position statements, and evaluation frameworks. This systematic review aimed to identify and characterize these documents, assess their methodological quality, and evaluate their applicability to AI tools supporting diagnostic imaging. **Methods**: PubMed, Scopus, Web of Science, Embase, and the Cochrane Library were searched in October 2025. English-language guidance documents first officially published or made available online between 2015 and 18 October 2025, including ahead-of-print articles, were eligible. Two reviewers independently screened records and assessed methodological quality using the Appraisal of Guidelines for Research and Evaluation II (AGREE II) with predefined project-specific interpretation guidance. The review was retrospectively registered in PROSPERO (CRD420261471153). **Results:** Sixteen guidance documents were included. Clarity of Presentation had the highest median AGREE II score (86.1%), whereas Rigour of Development had the lowest (35.4%). CLEAR, CLEAR-E3, the CLAIM 2024 Update and the European Society of Cardiovascular Radiology (ESCR) position statement were recommended for use; the remaining 12 documents were recommended with modifications. **Conclusions**: Radiology AI guidance is generally clear and applicable, but development methods are often insufficiently reported. No single imaging-specific document comprehensively addresses model development, validation, diagnostic workflow integration, and post-deployment monitoring; complementary guidance may therefore be required.

## 1. Introduction

Artificial intelligence (AI), particularly machine learning (ML) and deep learning (DL), is increasingly being evaluated and, in selected clinical settings, implemented in radiology as a task-specific decision support tool [1,2]. The prevailing clinical and regulatory landscape remains predominantly image-based: algorithms assist with the detection or localization of suspected abnormalities, segmentation of organs and lesions, quantitative measurements, and image reconstruction or enhancement [1]. These systems generally support rather than replace radiologist interpretation by highlighting findings or generating structured outputs for clinical verification.

Convolutional neural networks (CNNs) are widely used in medical image analysis for tasks such as lesion detection, classification, and pixel- or voxel-level segmentation. Their ability to learn relevant image features directly from imaging data has led to strong performance across a broad range of diagnostic applications. More recently, vision transformers (ViTs) have also been applied to detection, classification, and segmentation tasks. By using self-attention, these models can capture broader spatial relationships and global image context. Hybrid CNN–Transformer architectures combine the strengths of both approaches and have shown promising results in several medical imaging applications, although their performance still depends on factors such as dataset size, training strategy, and the specific clinical task [3,4]. Generative adversarial networks (GANs) have also been studied for image reconstruction and post-processing in low-dose CT. These models can transform -low dose computed tomography (CT) images to achieve image quality closer to that of standard-dose CT. This approach may reduce noise and artifacts and support CT examinations performed at lower radiation doses while maintaining diagnostically acceptable image quality [5,6].

Alongside these established applications, radiomics continues to be investigated, while generative AI, large language models (LLMs), and multimodal vision–language systems are expanding the research agenda. Proposed applications in radiology include report drafting and summarization, information extraction, patient-facing communication, and the joint analysis of images and text [7,8]. However, routine clinical deployment remains limited: a recent rapid review of multimodal LLMs in healthcare found that only a small minority of studies demonstrated real-world deployment, while workflow integration, governance, safety, and structured error analysis were rarely addressed [9]. These technologies should therefore be described as emerging rather than as standard components of current radiology practice.

Despite rapid technical progress, the evidence supporting radiology AI remains methodologically uneven. Reviews have identified a predominance of retrospective and frequently single-center studies, relatively small or selectively assembled datasets, limited prospective evaluation, and incomplete adherence to reporting standards [10,11]. External validation is often absent; when performed, model performance commonly decreases relative to that observed in development datasets, underscoring vulnerability to differences in patient populations, equipment, acquisition protocols, and clinical settings [12]. Additional threats include data leakage between training, validation, and test sets; inadequately defined reference standards; insufficient reporting of annotation, preprocessing, model selection, and hyperparameter tuning; and overinterpretation of performance observed during internal testing [13,14,15].

These methodological limitations have direct implications for diagnostic imaging in clinical practice, as the value of AI tools cannot be assessed solely on the basis of performance observed during model development and internal validation [12,16,17,18]. Evaluation of systems intended to support the diagnostic process should take into account their intended clinical use, characteristics of the target population, the quality of the reference standard, image acquisition and preprocessing procedures, and validation under conditions reflecting the intended use [15,16,17,18]. Clinical studies in radiology further indicate that AI may influence not only lesion detection performance and workflow organization, but also physicians’ diagnostic decisions [19,20]. The clinical consequences of false-positive and false-negative results are also important considerations when evaluating such systems [16]. Independent validation is particularly important because model performance may change when applied to external data, while systems intended for the same diagnostic task may differ in their achieved performance [12,21]. In this context, transparent reporting and evaluation standards are essential for determining whether observed model performance is reliable, generalizable, and potentially useful in real-world diagnostic practice [14,15,16,17,18].

Reporting and implementation guidance has consequently become essential for the reliable evaluation of diagnostic AI, as well as for reproducibility, critical appraisal, and safe clinical translation. Radiology-specific documents address different technologies and stages of AI system development, implementation, and use. CLAIM focuses on transparent reporting of medical imaging AI studies; CLEAR and CLEAR-E3 address radiomics; RIDGE focuses on the reproducibility and generalizability of segmentation models; PRIME and PRIME 2.0 address cardiovascular and multimodal AI evaluation; and iCARE addresses AI research in interventional radiology [14,15,16,22,23,24,25]. Broader healthcare guidance covers AI clinical-trial protocols and reports through SPIRIT-AI and CONSORT-AI, early live clinical evaluation through DECIDE-AI, prediction models through TRIPOD+AI, diagnostic-accuracy studies through STARD-AI, and trustworthy development, deployment, and monitoring across the AI lifecycle through FUTURE-AI [26,27,28,29,30,31]. These documents are complementary rather than interchangeable because they differ in their intended users, study designs, technological scope, methodological detail, and attention to implementation, governance, cybersecurity, and post-deployment monitoring.

This systematic review aimed to identify and characterize reporting guidelines, checklists, position statements, and evaluation frameworks for AI, ML, DL, and radiomics in radiology and medical imaging, as well as to assess their methodological quality and applicability in the context of the development, evaluation, and clinical implementation of tools supporting diagnostic imaging. We compared their scope, structure, intended users, methodological and data-reporting requirements, validation standards, and implementation-related content, and appraised their methodological quality using the Appraisal of Guidelines for Research and Evaluation II (AGREE II) instrument with predefined project-specific interpretation guidance [32]. We also assessed the extent to which the identified documents addressed elements relevant to the reliable use of AI in the diagnostic process, including requirements for reference standards, validation and generalizability, integration into clinical workflows, safety, and post-deployment monitoring, and whether any single document comprehensively covered all these stages.

## 2. Materials and Methods

This systematic review evaluated the methodological quality, reporting standards, and applicability of reporting guidelines, checklists, consensus statements, position papers, and evaluation frameworks for AI, ML, DL and radiomics in medical imaging. The review was reported in accordance with the Preferred Reporting Items for Systematic Reviews and Meta-Analyses (PRISMA) 2020 statement [33] (Supplementary File S1). The review was retrospectively registered in PROSPERO on 5 August 2026 (registration number: CRD420261471153).

### 2.1. Search Strategy and Selection Criteria

**Figure 1:**
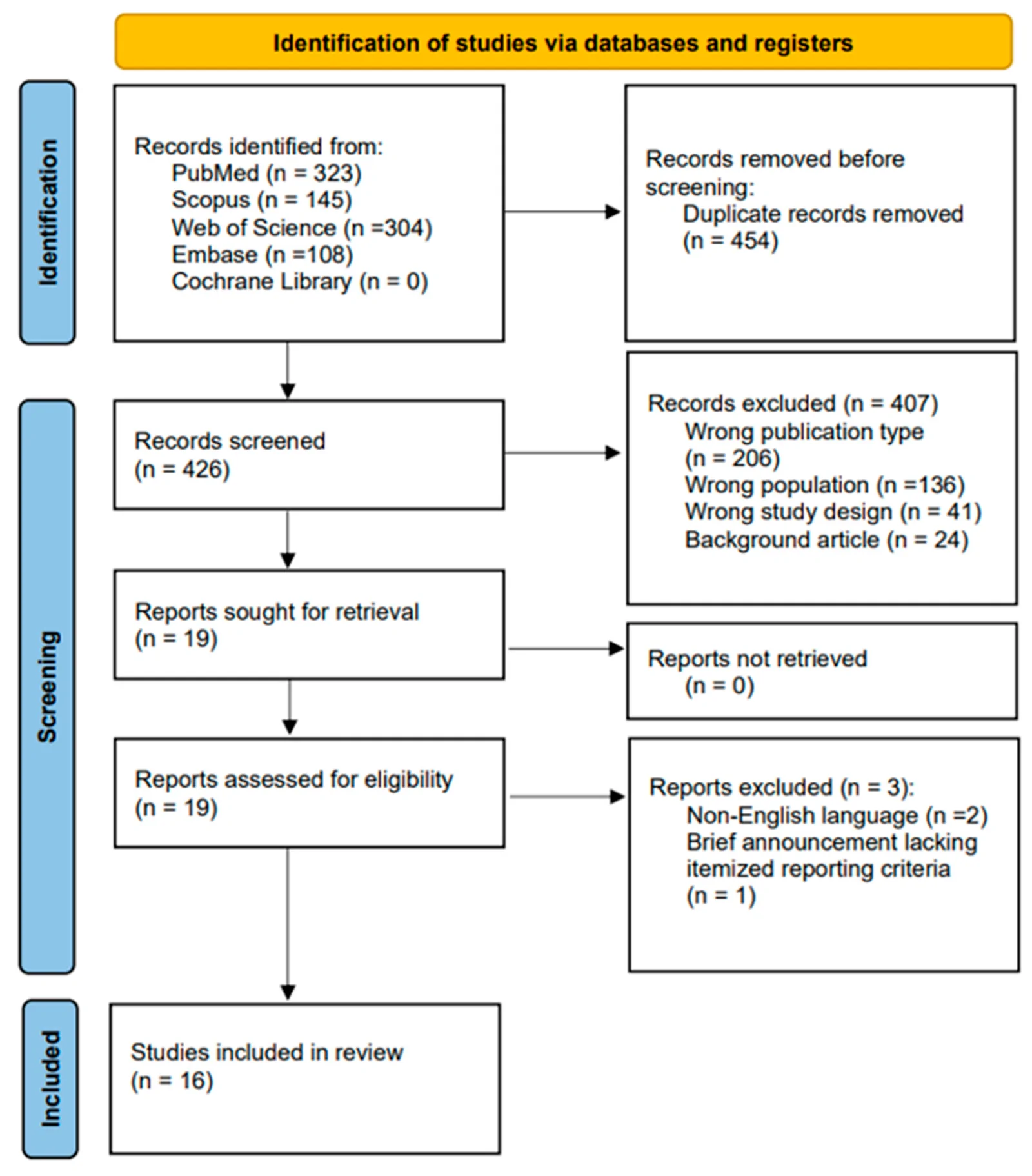
PRISMA flow diagram of the study selection process.

On 18 October 2025, a systematic search was conducted across PubMed, Scopus, Web of Science, Embase, and the Cochrane Library. The search strategy combined terms related to reporting standards such as reporting guideline, reporting standards, checklist, consensus statement, or position statement, with AI technologies including AI, ML, or DL, and radiology domains such as radiology, diagnostic imaging, or medical imaging. Full database queries are provided in Supplementary File S2. Literature retrieval was strictly confined to these five indexed electronic databases; gray literature searches, direct manual screening of professional medical imaging society websites, and formal backward or forward citation tracking of included reports were not performed. The initial search yielded 919 records. Applying filters for publication types such as articles or reviews and publication dates from 2015 to 2025, where available, reduced the pool to 880 records. Automated duplicate removal using Rayyan identified 680 potential duplicates, of which 454 were confirmed and deleted. Two independent reviewers screened the remaining 426 unique records based on titles and abstracts, excluding 407 records. Four inter-reviewer conflicts were resolved by consensus or through consultation with a third independent reviewer. Inter-rater agreement for screening, measured using Cohen’s kappa, was 0.890, indicating almost perfect agreement. Nineteen reports were sought for retrieval and successfully obtained. All 19 full-text reports were assessed for eligibility by two reviewers, and three publications were excluded (two due to non-English language [34,35], and one as a brief announcement lacking itemized reporting criteria [36]). Ultimately, 16 guidance documents met all eligibility criteria and were included in the synthesis. The selection workflow is shown in Figure 1.

### 2.2. Inclusion and Exclusion Criteria

Documents were eligible for inclusion if they met the following criteria:

- Reporting guidelines, checklists, consensus statements, position papers, or evaluation frameworks focused on AI, ML, DL, or radiomics in medical imaging.
- Published in English and first officially available online or in print between 2015 and 18 October 2025, including articles published ahead of print in peer-reviewed journals or endorsed by professional medical imaging societies, such as the European Society of Radiology (ESR), European Society of Medical Imaging Informatics (EuSoMII), American College of Cardiology (ACC), Canadian Association of Radiologists (CAR), Cardiovascular and Interventional Radiological Society of Europe (CIRSE), Radiological Society of North America (RSNA), or Asian Oceanian Society of Radiology (AOSR).

Eligibility was determined according to the date of first official publication or online availability. The bibliographic year subsequently assigned to a journal issue was used for reporting publication years in the extraction table and reference list.

We excluded empirical primary research articles evaluating an individual AI model without proposing a generalizable checklist or framework, narrative reviews lacking itemized reporting criteria, conference abstracts, editorials, and articles without full-text access. Broad biomedical and healthcare AI reporting guidelines, such as FUTURE-AI, TRIPOD+AI, STARD-AI, DECIDE-AI, SPIRIT-AI, and CONSORT-AI, were excluded from formal quality assessment. These documents were developed for cross-disciplinary clinical research or general healthcare applications rather than being specifically dedicated to radiology and medical imaging.

### 2.3. Data Extraction

Data were extracted independently by two reviewers using a standardized extraction form and compiled into a summary table provided in Supplementary File S3. Discrepancies between reviewers were resolved through discussion or by consulting a third independent reviewer. The extracted variables covered general study identification, including authors, publication year, country, journal, and digital object identifier. Additional extracted variables included clinical and technological scope such as imaging modalities, targeted clinical domains, AI model types, and intended target users. We also extracted framework structural characteristics like checklist acronyms, item counts, ground truth, or preprocessing requirements, as well as deployment factors including validation standards, cybersecurity, workflow impact, and reported limitations.

For the frequency-based synthesis, an element was considered addressed when the document contained an explicit recommendation, checklist item, criterion, or substantive instruction that required or recommended that the element be reported, assessed, defined, or performed. Background-only mentions without prescriptive or evaluative guidance were not counted.

### 2.4. Guideline Quality Assessment Using AGREE II

Because the included publications comprised reporting guidelines, checklists, position statements, and consensus papers rather than primary clinical trials, standard risk of bias tools were not applicable. Instead, methodological quality was assessed using the AGREE II instrument, with predefined project-specific interpretation guidance tailored to AI documents in medical imaging [32]. The original AGREE II structure was strictly maintained, including its 23 items across six domains, the 7-point rating scale, and the separate overall quality rating and recommendation. Content and item assignments were not modified. Project-specific interpretation guidance was developed to define how individual criteria applied to reporting guidelines, checklists, position statements, and evaluation frameworks. The complete project-specific working interpretation guidance is provided in Supplementary File S5. Before the formal assessment, the project-specific interpretation guidance was piloted on three guidance documents to calibrate its application between reviewers and resolve ambiguities in the interpretation of individual AGREE II items; no AGREE II items, domains, or scoring rules were modified. Two independent reviewers, designated as R1 and R2, evaluated each of the 16 included documents across all 23 AGREE II items using a standardized form. Each item was scored on a scale from 1 for strongly disagree or complete lack of criterion fulfillment to 7 for strongly agree or full criterion fulfillment. Discrepancies between R1 and R2 scores of two or more points were discussed to verify that both reviewers had interpreted and applied the AGREE II criteria correctly and consistently. These discussions were used to identify and clarify potential misunderstandings of the instrument rather than to eliminate genuine differences in judgment or to establish consensus item scores. Following these discussions, the original independent ratings of both reviewers were retained and used for subsequent AGREE II domain score calculations. The original item-level ratings of both reviewers are presented in Supplementary File S4.

### 2.5. Data Synthesis and AGREE II Scoring

Data were synthesized descriptively, and AGREE II domain scores were summarized using medians and ranges. Inter-rater reliability for the AGREE II assessment was evaluated separately for each domain using a two-way random-effects, absolute-agreement, single-measure intraclass correlation coefficient [ICC(2,1)] with 95% confidence intervals, based on the independent ratings of the two reviewers across the 16 included documents [37]. Analyses were performed in R version 4.6.1 using the irr package (version 0.85).

In accordance with the AGREE II user manual, domain performance was quantified by calculating Scaled Domain Scores expressed as percentages. The score for each domain represented the difference between the obtained score and the minimum possible score, divided by the difference between the maximum possible score and the minimum possible score, with the result multiplied by 100 percent. The obtained score represented the sum of ratings assigned by both reviewers to all items in that domain. Minimum and maximum possible scores were calculated based on the number of domain items, the participation of two reviewers, and the scale range from 1 to 7. Each domain was analyzed separately. No single composite percentage score for AGREE II was calculated, and no predetermined percentage thresholds were used to classify document quality. In addition, R1 and R2 independently assigned an overall quality rating on a 1–7 scale and a usage recommendation categorized as Yes, Yes with modifications, or No. Single final overall quality ratings and usage recommendations were subsequently established by consensus rather than mathematical aggregation. Individual reviewer scores are provided in Supplementary File S4.

Generative AI was used solely during manuscript preparation for language polishing, including minor grammatical, stylistic, and readability-related edits. It was not used for literature searching or screening, data extraction, methodological quality assessment, statistical analysis, or interpretation of the results.

## 3. Results

### 3.1. Clinical and Technological Scope

The analyzed publications could be divided into two groups. Eight publications had a general scope and broadly addressed the use of AI in radiology and medical imaging. This group included the CLAIM 2024 Update, CLEAR, CLEAR-E3, RIDGE, the checklist for reproducibility of DL in medical imaging, the radiology AI cybersecurity checklist, the guidelines of the Asian Oceanian Society of Radiology, and the CAR AI Specification and Evaluation Framework [14,15,17,22,24,38,39,40]. The remaining publications focused on specific clinical domains or practical aspects of AI implementation. These included prostate cancer detection using MRI, neuroradiology, cardiovascular imaging, pediatric radiology, and interventional radiology [16,18,23,25,41,42,43,44]. Some publications did not introduce entirely new tools but instead represented updates, extensions, or supplementary documents to previously developed checklists. These included the CLAIM 2024 Update [15], CLEAR-E3 as an extension of CLEAR [22,24], and PRIME 2.0 as an update to the PRIME checklist [16,23].

With regard to imaging modalities, 15 of the 16 publications were not limited to a single imaging technique [14,15,16,17,22,23,24,25,38,39,40,41,42,43,44], whereas one publication focused exclusively on prostate magnetic resonance imaging (MRI) [18]. Collectively, the multimodal publications covered CT, MRI, radiography, ultrasonography, echocardiography, mammography, and angiography. Publications concerning cardiovascular imaging primarily addressed CT, MRI, and echocardiography [16,23,42]. PRIME 2.0 additionally expanded the scope of included data to angiography, nuclear imaging, and signal-based data such as electrocardiogram (ECG) [16]. One document extended beyond conventional imaging data to include text and audio data [25].

All analyzed publications addressed AI applications in radiology or medical imaging, although their technological scope varied. ML methods were included in 15 documents [14,15,16,17,18,22,23,24,25,38,40,41,42,43,44], whereas DL or related methods, such as CNNs, vision transformers, and deep residual networks, were addressed in 12 of the 16 publications [14,15,16,22,23,24,25,38,39,40,41,43]. Radiomics methods were discussed in two publications [22,24], as were large language models and generative AI [16,25]. Natural language processing (NLP)-related issues were addressed in one publication [17], while software as a medical device and AI tools integrated with medical hardware were discussed in one publication [44].

### 3.2. Structure and Intended Use of the Analyzed Tools

The structural analysis of the identified guidance documents revealed considerable variation in their length, organization, and target audiences. The tools ranged from concise 10–11-item checklists or sets of criteria, such as R-AI-DIOLOGY, the PI-RADS AI Model Checklist, the ESCR position statement, and the implementation guide for AI in interventional radiology [18,41,42,44], to comprehensive evaluation frameworks comprising several dozen items, including 44 items in the CLAIM 2024 Update [15], 55 items in iCARE [25], and the 58-item CLEAR checklist, together with its CLEAR-E3 explanation and elaboration document [22,24]. PRIME and PRIME 2.0 were distinguished by a domain-based structure comprising seven major areas of assessment [16,23].

Regarding target audiences, six documents were directed specifically at authors, researchers, and reviewers to support standardized reporting of scientific studies [14,15,22,24,25,39]. Four publications focused on the evaluation and implementation of ready-to-use AI software in clinical practice and were intended for hospital managers, regulators, decision-makers, IT professionals, and clinical teams [17,40,41,44]. The remaining documents had broader or mixed audiences, including researchers and model developers as well as physicians and other clinical users. A detailed overview of the structure, number of items, and primary purpose of the analyzed documents is presented in Table 1.

### 3.3. Quality Assessment of the Analyzed Documents

The methodological quality assessment of the analyzed documents, performed using the AGREE II instrument, demonstrated considerable variation in domain scores across publications. The highest median scaled domain score was obtained for Clarity of Presentation (D4), at 86.1%, with scores ranging from 77.8% to 88.9%, whereas the lowest median score was observed for Rigour of Development (D3), at 35.4%, with scores ranging from 22.9% to 54.2%. The median score for Scope and Purpose (D1) was 72.2% (range: 61.1–94.4%), that for Stakeholder Involvement (D2) was 58.3% (range: 44.4–63.9%), that for Applicability (D5) was 74.0% (range: 62.5–87.5%), and that for Editorial Independence (D6) was 75.0% (range: 33.3–91.7%).

Inter-rater reliability varied across AGREE II domains. ICC(2,1) values were 0.78 for Scope and Purpose, 0.78 for Stakeholder Involvement, 0.78 for Rigour of Development, 0.31 for Clarity of Presentation, 0.72 for Applicability, and 0.36 for Editorial Independence. The corresponding 95% confidence intervals are provided in Supplementary File S4.

The highest overall quality rating of 6 points was assigned to CLEAR, CLEAR-E3, the CLAIM 2024 Update, and the ESCR position statement; all four documents received a recommendation of “Yes” [15,22,24,42]. Eleven publications received an overall quality rating of 5 points, whereas the radiology AI cybersecurity checklist was the only document to receive a rating of 4 points [40]. Overall, 12 publications received a recommendation of “Yes, with modifications,” and four received a recommendation of “Yes.” No document received a recommendation of “No.” Detailed results are presented in Table 2.

### 3.4. Methodological Requirements and Data Standards

With regard to methodological rigor and data management standards, the included documents emphasized the need for precise reporting of annotation procedures and image transformations. Requirements related to defining the reference standard or describing the annotation process were included in 13 of the 16 publications [14,15,16,17,18,22,24,25,38,39,41,42,43]. The CLAIM 2024 Update adopted the term “reference standard” in place of “ground truth” and required reporting of annotator qualifications and training, as well as measures of intra- and inter-rater variability [15]. RIDGE specified the need to involve multiple experts and to describe methods for resolving disagreements [14]; iCARE required reporting whether IR clinicians or other healthcare professionals were involved in the annotation process and, if not, an explanation of why their involvement was not possible or necessary [25], whereas the CAR Framework recommended the use of multi-expert consensus or verification based on clinical and pathological outcomes [17].

Requirements concerning image preprocessing or the technical preparation of imaging data were included in 12 of the 16 publications [14,15,16,17,18,22,23,24,25,39,41,43]. The CLAIM 2024 Update required detailed reporting of normalization, resampling, windowing, binarization, de-identification, and the handling of missing data [15]. iCARE required the reporting of bias-mitigation techniques and data masking [25], whereas the CAR Framework assigned responsibility to developers by requiring them to specify spatial requirements, slice thickness, and vascular phases [17]. Detailed methodological requirements for model development were presented in the DL reproducibility checklist, RIDGE, and the CLAIM 2024 Update. These included descriptions of the algorithm type, model architecture, training parameters, optimization procedures, and criteria used to select the final model [14,15,39].

### 3.5. Validation, Diagnostic Applicability, and Clinical Workflow

Given the nature of the analyzed publications, which primarily comprised guidelines, checklists, conceptual frameworks, and expert position statements, none presented direct clinical validation of a new AI model or assessed its impact on diagnostic accuracy. Nevertheless, external or independent testing or validation of AI models was addressed in 13 publications [14,15,16,17,18,22,23,24,25,38,41,42,43]. The remaining three documents did not specify clear criteria for evaluating a model using independent data [39,40,44].

With regard to workflow, the documents emphasized the need to report the intended clinical role of the algorithm, translational barriers, and computational burden [14,15]. The CAR Framework prioritized the assessment of AI-related effects on urgent findings notifications, scanner utilization, report turnaround time, and staff satisfaction [17]. In addition, practically oriented guidance highlighted the need to assess risks associated with continuous model learning, concept drift, and the degradation of software performance over time [44]. A summary of the main methodological, validation-related, and clinical implementation domains identified across the analyzed documents is presented in Table 3.

### 3.6. Synthesis of the Main Directions of the Analyzed Documents

The analysis identified two broad and partly overlapping document profiles. The first comprised tools that emphasized scientific transparency and reproducibility through reporting requirements related to data partitioning, reference standards, preprocessing, model development, and validation [14,15,16,18,22,23,24,25,39,42,43]. The second comprised evaluation frameworks focused on the implementation and governance of AI in clinical practice. These documents extended beyond methodological considerations and also addressed non-medical factors, including risks related to autonomous device operation, the financial feasibility of implementation, pricing models such as subscriptions and licensing, cybersecurity procedures, and the auditability of commercial software code [17,38,40,41,44].

## 4. Discussion

This review focused on 16 documents addressing the reporting, evaluation, and implementation of AI in radiology. The analysis demonstrated considerable variation in their scope and intended use. The AGREE II assessment indicated that the recommendations were generally presented clearly and transparently, whereas the methods used to develop them were described in less detail. At the same time, none of the analyzed documents comprehensively addressed all aspects of AI tool development, validation, clinical implementation, and post-deployment monitoring. This was reflected in the final assessments, as most documents were considered useful but were recommended for use with modifications.

### 4.1. Methodological Quality of the Included Guidance Documents

A notable strength of the analyzed publications was the clarity with which the recommendations were presented. In most documents, the main principles were formulated clearly and presented in a structured and easily identifiable manner. This was reflected in the highest score obtained for Domain 4, Clarity of Presentation, with a median of 86.1% and scores ranging from 77.8% to 88.9%. Most documents were also characterized by a clearly defined purpose and scope of application. The median score for Domain 1, Scope and Purpose, was 72.2%, with a range from 61.1% to 94.4%.

Relatively high scores were also obtained for Domain 5, Applicability. The median was 74.0%, while scores across individual documents ranged from 62.5% to 87.5%. This was associated with the inclusion of practical aspects of guideline use, such as implementation barriers, technical requirements, effects on clinical workflows, and the need to monitor the performance of AI tools.

Among the 16 analyzed publications, only four received a final recommendation of “Yes” [15,22,24,42]. CLEAR [22] achieved the highest scores for Rigour of Development (D3: 54.2%) and Editorial Independence (D6: 91.7%), whereas the CLAIM 2024 Update [15] obtained the highest score for Stakeholder Involvement (D2: 63.9%). The ESCR position statement [42] combined high clarity of presentation (D4: 88.9%) with strong applicability (D5: 81.2%). CLEAR-E3 [24] showed relatively balanced performance across all domains, with scores ranging from 49.0% to 83.3%. These findings suggest that the high final ratings of these documents did not result from particularly strong performance in only one domain, but rather from maintaining relatively consistent quality across the assessed domains.

The main limitations of the analyzed documents concerned the methods used in their development. Domain D3, which assessed Rigour of Development, covered the methods used to search for and select evidence, the process of formulating recommendations, expert involvement in reaching consensus, external review, and procedures for future updates to the documents. It received the lowest score among all assessed domains, with a median of 35.4% and scores ranging from 22.9% to 54.2%. These findings were associated with insufficiently detailed reporting of the successive stages of guideline development, which made it more difficult to assess the systematic nature of evidence collection and the transparency of the methodological process. This does not necessarily indicate that the recommendations themselves were of low value, but rather suggests inadequate reporting of how they were developed.

Despite the limitations identified, 12 of the 16 publications received a recommendation of “Yes, with modifications,” indicating that they could be applied provided that their limitations were taken into account. The main areas requiring further development included greater transparency of the development process, broader involvement of different stakeholder groups, and more complete reporting of editorial independence. None of the analyzed publications received a recommendation of “No.” This suggests that their main limitation was not a lack of usefulness, but rather incomplete adherence to the assessed quality criteria.

### 4.2. Comparison with Selected AI Guidance in Other Image-Based Medical Fields

To place the findings of this review in a broader context, the analyzed radiology guidance documents were compared with selected documents developed for other image-based medical fields, particularly endoscopy, as well as with a general medical framework for image annotation, illustrated using digital pathology. The analyzed radiology documents frequently addressed technical issues specific to imaging data, including the definition of a reference standard and annotation procedures [14,15,16,17,18,22,24,25,38,39,41,42,43], image preprocessing or other forms of technical data preparation [14,15,16,17,18,22,23,24,25,39,41,43], and external or independent model testing [14,15,16,17,18,22,23,24,25,38,41,42,43]. Some documents also addressed technical imaging parameters [15,17], cybersecurity [40], workflow impact [14,15,17,44], and post-deployment model monitoring [44].

In gastroenterology, the QUAIDE framework focused on preclinical AI studies in endoscopy and included 18 recommendations addressing, among other areas, data acquisition and annotation, experimental setup, algorithm architecture, and the presentation and interpretation of results. The recommendations were developed using a formal Delphi process involving an international, multidisciplinary panel of 32 experts [45]. The publication provided a detailed description of the anonymous and iterative voting process, the predefined consensus threshold, and the procedures for modifying or removing statements. QUAIDE may therefore serve as an example of more detailed reporting of the consensus-development process, whereas this level of methodological detail was not consistently reported across all radiology guidance documents included in the present review.

In contrast, the Japan Gastroenterological Endoscopy Society position statement focused on practical aspects of AI use in endoscopy, including cost-effectiveness, potential adverse clinical consequences, medical safety, and legal responsibility associated with the use of AI tools [46]. Similar issues were also addressed in some of the analyzed radiology guidance documents, although they were not covered consistently across publications.

A more focused approach to the development and evaluation of annotation datasets was presented in CLEARR-AI. This framework was designed for human-interpreted medical images, and its application was illustrated using digital pathology as an example. CLEARR-AI recommends reporting, among other elements, the selection and qualifications of annotators, the instructions and training materials provided to them, the data dictionaries used, methods for adjudicating or verifying expert annotations, and quality-control procedures for both the annotation study and the resulting dataset [47]. Annotation-related issues were frequently addressed in the analyzed radiology guidance documents; however, they usually represented only one of several evaluated domains, whereas CLEARR-AI focused specifically on the reproducible reporting of annotation dataset development and evaluation.

Taken together, these examples suggest that the analyzed radiology guidance documents cover a broad range of issues and account for the technical characteristics of imaging data. Selected documents from other medical fields, however, addressed individual areas in greater detail, including the guideline development process, the organization and quality control of annotations, and the economic, legal, and clinical implications of AI implementation. These elements were also present in some of the analyzed radiology documents, but they were not addressed consistently or combined within a single comprehensive document.

### 4.3. Clinical Implications for Diagnostic Imaging

The reliability of AI tools used in diagnostic imaging depends not only on reported performance metrics, but also on how the reference standard is defined, how imaging data are prepared, and how validation is performed. In the present review, requirements related to the reference standard or annotation process were addressed in 13 of 16 documents, whereas 12 documents included requirements concerning image preprocessing or other forms of technical data preparation. These elements are relevant not only to reproducibility but also to the interpretation of observed diagnostic performance, because the quality of the reference data, image preparation procedures, and characteristics of the target population may affect model performance [12,15,16,17,18]. Similarly, the finding that 13 of 16 documents addressed external validation or independent testing highlights the importance of assessing generalizability before implementation in clinical practice [12].

Clinical studies in radiology illustrate why these requirements matter. In the MASAI trial, AI-supported mammography screening affected both cancer detection and screen-reading workload, indicating that evaluation of diagnostic AI should consider workflow effects alongside detection performance [19]. In chest radiography, the way AI-generated information is presented has been shown to influence physicians’ diagnostic performance and trust in AI [20]. Independent comparisons of commercial AI systems designed for the same diagnostic tasks have also demonstrated differences in performance, further supporting the need for representative external validation before clinical use [21].

Another potential clinical application of AI is the opportunistic assessment of findings captured on imaging performed for a different primary indication. For example, low-dose chest CT acquired for lung cancer screening may also be used for automated detection and quantification of coronary artery calcification and emphysema. Such approaches may provide additional information on cardiovascular and respiratory disease without requiring additional imaging or radiation exposure [48]. These secondary applications further highlight the need to clearly define and report the intended use, validation strategy, and performance of AI tools for each clinical task.

At the same time, our synthesis shows that requirements relevant to the reliable clinical use of AI are distributed across different guidance documents. Some focus primarily on reporting, data handling, model development, and validation [14,15,16,18,22,23,24,25,39,42,43], whereas others place greater emphasis on clinical implementation, workflow impact, cybersecurity, operational risk, and post-deployment monitoring [17,38,40,41,44]. Among the evaluated guidance documents specific to radiology and medical imaging that met our inclusion criteria, no single document comprehensively covered the entire pathway from model development and validation to integration into the diagnostic workflow and long-term post-deployment oversight. From a clinical perspective, this suggests that evaluating AI tools intended to support diagnostic imaging may require the combined use of several complementary guidance documents.

### 4.4. Practical Implications and Future Directions

The findings of this review indicate that the choice of guidance document should depend on its intended purpose. Checklists focused on research reporting, such as CLEAR, CLEAR-E3, PRIME, PRIME 2.0, RIDGE, and the CLAIM 2024 Update, may support authors and reviewers in assessing methodological clarity [14,15,16,22,23,24]. However, they may need to be complemented by documents focused on the validation, implementation, or monitoring of AI tools in clinical practice, such as R-AI-DIOLOGY, the radiology AI cybersecurity checklist, the CAR AI Framework, and the implementation guide for interventional radiology [17,40,41,44].

In some situations, the simultaneous use of several complementary documents may therefore be appropriate. The main advantages and limitations associated with the use of AI reporting guidelines in radiology are summarized in Table 4.

Future guidance and updates to existing documents should aim to integrate requirements for model development and reporting with the assessment of clinical utility, workflow impact, safety, and post-deployment oversight. At the institutional level, dedicated multidisciplinary AI governance committees may play an important role in the safe implementation of clinical algorithms. Such committees may support the selection of AI tools, assess whether their intended use is appropriate for the local clinical setting, and oversee validation using representative institutional data before deployment. Their role should also extend beyond initial implementation to continued monitoring of model performance, including changes related to patient populations, imaging equipment, acquisition protocols, software updates, and workflow. Multidisciplinary involvement of clinicians, radiologists, informatics specialists, data scientists, and quality and safety representatives may help ensure that decisions regarding adoption, modification, or withdrawal of AI tools are based on both technical performance and clinical relevance [2,49].

Greater emphasis should also be placed on the transparent description of the process used to develop recommendations, the involvement of different stakeholder groups, funding sources and conflicts of interest, and procedures for regular document updates. These issues are becoming particularly important given the rapid development of generative AI, large language models, multimodal systems, and tools that may continue to evolve after deployment [16,25,44].

### 4.5. Limitations

This review has several limitations. The AGREE II instrument was applied using project-specific interpretation guidance to assess documents of different types, including checklists, expert position statements, practical guides, and evaluation frameworks. Consequently, not all AGREE II items were equally applicable to every included document. Despite independent assessment by two reviewers, some ratings may have remained dependent on individual interpretation of the criteria. Furthermore, our search relied exclusively on five indexed electronic bibliographic databases without manual searches of individual medical imaging society websites or reference lists. Consequently, non-indexed society-endorsed recommendations, institutional consensus statements, or clinical practice white papers may not have been captured.

The review also included updates and extensions of previously developed tools, which may have resulted in partial overlap in the evaluated content. In addition, the review was limited to English-language publications with available full texts. Finally, the rapid development of AI in medical imaging, particularly in radiology, means that new or updated guidance documents may have been published after the literature search was completed.

## 5. Conclusions

The included documents addressing AI in radiology varied in scope, intended use, and methodological quality. Although the recommendations were generally presented clearly and had practical value, the processes used to develop them were often described in insufficient detail. Most documents were considered useful but were recommended for use with modifications. However, none of the included frameworks specific to radiology comprehensively addressed all stages from model development and validation to integration into the diagnostic workflow and post-deployment monitoring. For AI tools intended to support diagnostic imaging, reliable evaluation therefore requires not only transparent reporting of model performance, but also appropriate reference standards, representative validation, consideration of workflow effects and safety, and continued monitoring in clinical use. The choice of an appropriate guidance document should reflect the intended purpose and may require the simultaneous use of several complementary documents. Future guidance should integrate methodological reporting requirements with the assessment of diagnostic reliability, clinical utility, safety, implementation, and post-deployment monitoring of AI tools used in diagnostic imaging.

## Figures and Tables

**Table 1:** Structure and intended use of included AI guidelines, checklists, and evaluation frameworks in radiology.

Document/Tool	Structure	Intended Users	Main Intended Use
AOSR position statements [38]	Position statement	Radiologists and healthcare institutions	Supporting ethical AI adoption, governance, and integration into radiology workflows
CAR AI Specification and Evaluation Framework [17]	24 items/framework	Healthcare decision-makers, regulators, and radiologists	Pre-adoption specification and evaluation of clinical AI software
CLAIM 2024 Update [15]	44 items	Authors and reviewers	Transparent and reproducible reporting of medical imaging AI studies
CLEAR [22]	58 items; CLEAR-S 43 items	Authors and reviewers	Reporting and appraisal of radiomics research
CLEAR-E3 [24]	Explanation and elaboration for 58 CLEAR items	Authors, editors, and reviewers	Providing detailed guidance and examples for applying CLEAR
Checklist for reproducibility of DL in medical imaging [39]	26 items	Researchers and authors	Reproducible reporting of deep-learning model development in medical imaging
ESCR position statement [42]	11 quality criteria	Cardiovascular imaging specialists and machine-learning researchers	Design, reporting, interpretation, and quality assessment of cardiovascular imaging ML studies
iCARE [25]	55 items	Interventional radiology researchers and reviewers	Standardized reporting and evaluation of AI research in interventional radiology
Introductory guide to AI in IR: implementation [44]	11-point checklist	Interventional radiologists and clinical implementation teams	Safe implementation, workflow integration, and risk assessment of AI tools
Pediatric radiology AI reporting guidelines [43]	Review of multiple guidelines	Pediatric radiologists, researchers, and reviewers	Supporting the application of AI reporting guidance in pediatric radiology
PI-RADS AI Model Checklist [18]	11 items	Researchers, radiologists, and AI developers	Development and reporting of AI models for clinically significant prostate cancer detection
PRIME [23]	7 domains	Investigators, data scientists, authors, reviewers, and editors	Standardized evaluation and reporting of cardiovascular imaging ML studies
PRIME 2.0 [16]	7 domains	Researchers, clinicians, reviewers, and editors	Reporting and evaluation of contemporary multimodal AI studies in cardiovascular imaging
R-AI-DIOLOGY [41]	10 checkpoints	Clinical neuroradiologists	Practical evaluation of AI tools before clinical implementation
Radiology AI cybersecurity checklist [40]	Checklist within CIA framework	Radiology practices, IT teams, and healthcare institutions	Cybersecurity assessment before and during AI deployment
RIDGE [14]	46 items	Researchers and reviewers	Assessment and reporting of reproducibility and generalizability in DL segmentation studies

**Table 2:** Standardized AGREE II domain scores, overall quality ratings, and final recommendations for the included guidance documents.

Guidance Document	AGREE II Standardized Domain Score (%) ^1,2^						Overall Assessment	
	D1	D2	D3	D4	D5	D6	Quality	Recommendation
AOSR position statements [38]	61.1	44.4	37.5	83.3	70.8	75.0	5	Yes, with modifications
CAR AI Framework [17]	72.2	58.3	30.2	88.9	87.5	70.8	5	Yes, with modifications
CLAIM 2024 Update [15]	72.2	63.9	44.8	86.1	72.9	79.2	6	Yes
CLEAR [22]	72.2	61.1	54.2	77.8	70.8	91.7	6	Yes
CLEAR-E3 [24]	72.2	58.3	49.0	83.3	70.8	79.2	6	Yes
DL reproducibility checklist [39]	72.2	55.6	32.3	77.8	62.5	75.0	5	Yes, with modifications
ESCR position statement [42]	72.2	58.3	44.8	88.9	81.2	83.3	6	Yes
iCARE [25]	72.2	58.3	37.5	86.1	79.2	75.0	5	Yes, with modifications
Introductory guide to AI in IR [44]	66.7	52.8	22.9	86.1	81.2	79.2	5	Yes, with modifications
Pediatric radiology AI guide [43]	72.2	52.8	24.0	83.3	66.7	87.5	5	Yes, with modifications
PI-RADS AI Model Checklist [18]	94.4	55.6	47.9	83.3	70.8	33.3	5	Yes, with modifications
PRIME [23]	72.2	61.1	33.3	86.1	68.8	62.5	5	Yes, with modifications
PRIME 2.0 [16]	72.2	61.1	37.5	88.9	87.5	41.7	5	Yes, with modifications
R-AI-DIOLOGY [41]	72.2	50.0	22.9	88.9	75.0	75.0	5	Yes, with modifications
Radiology AI cybersecurity checklist [40]	66.7	47.2	22.9	88.9	79.2	50.0	4	Yes, with modifications
RIDGE [14]	72.2	61.1	29.2	88.9	75.0	66.7	5	Yes, with modifications

^1^ D1, Scope and Purpose; D2, Stakeholder Involvement; D3, Rigour of Development; D4, Clarity of Presentation; D5, Applicability; D6, Editorial Independence. Domain scores are presented as standardized percentages. ^2^ Overall quality was rated separately on a 1–7 scale and was not calculated as the mean of the six domains. Final overall quality ratings and recommendations were agreed by consensus.

**Table 3:** Summary of key methodological, validation- and implementation-related domains identified across included documents.

Domain	Summary of Extracted/Reviewed Requirements	Main Documents
Reference standard and annotation	Requirements to define the reference standard and describe annotation methodology, annotator expertise or training, intra-/inter-rater variability, expert consensus and discrepancy resolution.	[14,15,17,25]
Image data handling and preprocessing	Reporting of image processing steps, including normalization, resampling, windowing, binarization, de-identification, handling of missing data, radiomics processing and technical image requirements.	[15,17,22,24,25]
Model development and reproducibility	Description of algorithm type, model architecture, training parameters, optimization, model selection and reproducibility of DL or radiomics workflows.	[14,15,22,24,39]
Validation and diagnostic impact	External or clinical validation is recommended or discussed; the included documents did not provide independent validation of new AI models or direct assessment of diagnostic accuracy impact.	[14,15,16,17,23]
Workflow, safety and clinical use	Evaluation of clinical role, workflow impact, computational burden, cybersecurity, operational risk, model drift or depreciation and monitoring over time.	[14,15,17,38,40,44]

**Table 4:** Advantages and limitations of using AI reporting guidelines in radiology.

Aspect	Advantages	Limitations	Main Documents
Reporting transparency	Provides a structured framework for reporting study design, datasets, model development, and performance.	Adherence to a reporting checklist does not itself ensure high methodological quality.	[14,15,22,24,39]
Reproducibility	Encourages detailed reporting of preprocessing, model architecture, training procedures, reference standards, and annotation methods.	The level of technical detail required varies between guidelines, and important information may still be omitted.	[14,15,22,24,39]
Validation and generalizability	Promotes reporting of external or independent validation and characteristics of the target population.	Validation requirements are not consistently addressed across all guidelines, and adherence to a guideline does not itself ensure model generalizability.	[14,15,16,17,18,22,23,24,25]
Standardization	Facilitates more consistent reporting between studies and may improve comparison and critical appraisal of AI research.	Existing guidelines differ in scope, terminology, and requirements, which may make selection of the most appropriate document difficult.	[14,15,16,17,18,22,23,24,25,39,42,43]
Clinical applicability	Some frameworks address intended clinical use, workflow integration, safety, and practical implementation of AI tools.	Many guidelines primarily focus on research reporting and provide limited guidance on real-world implementation or clinical effectiveness.	[17,38,41,44]
AI lifecycle and monitoring	Selected documents address cybersecurity, operational risks, model drift, and post-deployment monitoring.	These aspects are not consistently covered, and no single radiology-specific guideline identified in this review addressed the entire AI lifecycle.	[17,38,40,41,44]
Guideline development	Expert-developed recommendations can support authors, reviewers, developers, and clinical users.	Development methods, stakeholder involvement, external review, and updating procedures are often insufficiently reported.	[15,22,24,42]
Practical use	Checklists can help authors and reviewers identify missing methodological or reporting elements before publication.	Using several complementary guidelines may increase complexity and reporting burden, particularly when requirements overlap.	[14,15,16,17,22,23,24,25,39,40,41,42,43,44]

## Data Availability

The data generated and analyzed during this study are provided in the article and its Supplementary Materials.

## Supplementary Materials

The following supporting information can be downloaded at https://www.mdpi.com/article/10.3390/diagnostics16183054/s1. Supplementary File S1: PRISMA 2020 Checklist; Supplementary File S2: Search strategy; Supplementary File S3: Complete data extraction; Supplementary File S4: Detailed AGREE II assessment of the included guidance documents; Supplementary File S5: Project-specific working interpretation guidance for the AGREE II assessment. Refs. [14,15,16,17,18,22,23,24,25,32,33,38,39,40,41,42,43,44] have been cited in the Supplementary Materials.
