# Supplementary material for: Artificial Intelligence Reporting Guidelines in Radiology: A Systematic Review
Source: Diagnostics (Basel). 2026 Sep 20;16(18):3054. doi: 10.3390/diagnostics16183054 (PMC13605318; doi:10.3390/diagnostics16183054)
Supplement: Supplementary file 1 [file diagnostics-16-03054-s001.zip › Supplementary File S2. Search strategy.pdf]

Search strategy (18 October 2025)

Search string:
("reporting guideline" OR "reporting standards" OR "checklist" OR "consensus statement" OR "position statement") AND ("artificial intelligence" OR "AI" OR "machine learning" OR "deep learning") AND ("radiology" OR "diagnostic imaging" OR "medical imaging")

PubMed:
("reporting guideline" OR "reporting standards" OR "checklist" OR "consensus statement" OR "position statement") AND ("Artificial Intelligence" OR "AI" OR "Machine learning" OR "deep learning") AND ("Radiology" OR "Diagnostic Imaging" OR "Medical imaging")

324 -> Filters: in the last 10 years -> 323

323:
https://pubmed.ncbi.nlm.nih.gov/?term=%28%22reporting+guideline%22+OR+%22reporting+standards%22+OR+%22checklist%22+OR+%22consensus+statement%22+OR+%22position+statement%22%29+AND+%28%22Artificial+Intelligence%22+OR+%22AI%22+or+%22Machine+learning%22+OR+%22deep+learning%22%29+AND+%28%22Radiology%22+OR+%22Diagnostic+Imaging%22+OR+%22Medical+imaging%22%29&filter=datesearch.y_10

Scopus:
(TITLE-ABS("reporting guideline" OR "reporting standards" OR "checklist" OR "consensus statement" OR "position statement")) AND (TITLE-ABS("Artificial Intelligence" OR "Machine Learning" OR "Deep Learning")) AND (TITLE-ABS("Radiology" OR "Diagnostic Imaging" OR "Medical Imaging"))

155 -> Filters: in the last 10 years, article, conference paper, review -> 145

145:
https://www.scopus.com/results/results.uri?sort=plf-f&src=s&sid=86bdd31c9339252df7b4ca0289354f1a&sot=a&sdt=cl&sl=281&s=%28TITLE-ABS%28%22reporting+guideline%22+OR+%22reporting+standards%22+OR+%22checklist%22+OR+%22consensus+statement%22+OR+%22position+statement%22%29%29+AND+%28TITLE-ABS%28%22Artificial+Intelligence%22+OR+%22Machine+Learning%22+OR+%22Deep+Learning%22%29%29+AND+%28TITLE-ABS%28%22Radiology%22+OR+%22Diagnostic+Imaging%22+OR+%22Medical+Imaging%22%29%29&origin=resultslist&editSaveSearch=&txGid=a8e1a6a161587677c23b6c7534d34eed&sessionSearchId=86bdd31c9339252df7b4ca0289354f1a&limit=10&cluster=scosubtype%2C%22cp%22%2Ct%2C%22ar%22%2Ct%2C%22re%22%2Ct

Web of Science:
TS=("reporting guideline" OR "reporting standards" OR "checklist" OR "consensus statement" OR "position statement") AND TS=("artificial intelligence" OR "machine learning" OR "deep learning") AND TS=("radiology" OR "diagnostic imaging" OR "medical imaging")

304 -> Filters: no -> 304

https://www.webofscience.com/wos/alldb/summary/ad354c95-74d7-43ba-ba61-38388bd3b484-0182017939/relevance/1

Embase:
('reporting guideline':ab,ti OR 'reporting standard':ab,ti OR 'checklist':ab,ti OR 'consensus statement':ab,ti OR 'position statement':ab,ti) AND ('artificial intelligence'/exp OR 'artificial intelligence':ab,ti OR 'machine learning':ab,ti OR 'deep learning':ab,ti) AND ('radiology'/exp OR 'diagnostic imaging':ab,ti OR 'radiology':ab,ti OR 'medical imaging':ab,ti)

136 -> Filters: article, review -> 108

https://www.embase.com/#advancedSearch/resultspage/history.2/page.1/25.items/orderby.date/source
Cochrane:
("reporting guideline" OR "reporting standards" OR checklist OR "consensus statement" OR "position statement") AND ("artificial intelligence" OR "machine learning" OR "deep learning") AND ("radiology" OR "diagnostic imaging" OR "medical imaging") :ti,ab,kw

0 results

Results of search summary:
All articles searched: 919
All articles searched after filters: 880
Duplicates automatically detected: 680
Duplicates deleted: 454
First scanning (title/abstract screening): 426
The conflicts between researchers: 4
Researcher 1: excluded 409, included 17
Researcher 2: excluded 405, included 21
Articles accepted for retrieval: 19
Articles removed after full-text assessment: 3; 2 - non-English, 1 - brief announcement lacking itemized reporting criteria.
Articles rejected: 407
Articles included in study: 16

Results:
Cohen's k: 0.890

Almost perfect agreement
