# Supplementary material for: Artificial Intelligence Reporting Guidelines in Radiology: A Systematic Review
Source: Diagnostics (Basel). 2026 Sep 20;16(18):3054. doi: 10.3390/diagnostics16183054 (PMC13605318; doi:10.3390/diagnostics16183054)
Supplement: Supplementary file 1 [file diagnostics-16-03054-s001.zip › Supplementary File S4. Detailed AGREE II assessment-edit of the included guidance documents.pdf]

Two reviewers independently appraised the 16 included guidance publications using the original 23-item AGREE II instrument with project-specific interpretation guidance. Item-level ratings are presented as R1/R2. Discrepancies of at least two points were discussed; the original independent ratings were retained for calculation of standardized domain scores. Final overall quality ratings and recommendations were agreed by consensus.

Table S1A. Item-level AGREE II ratings for the included guidance publications (items 1–12).

Guidance document	I1	I2	I3	I4	I5	I6	I7	I8	I9	I10	I11	I12
PI-RADS AI Model Checklist	7/7	7/7	6/6	6/6	1/1	6/6	2/2	4/4	5/5	6/6	5/5	5/5
AOSR position statements	6/6	5/5	3/3	4/4	1/1	6/6	1/1	1/1	2/2	5/5	6/6	6/6
CLEAR	7/7	6/6	3/3	6/6	1/1	7/7	2/2	2/2	2/2	7/7	5/5	6/6
R-AI-DIOLOGY	7/7	6/6	3/3	4/4	1/1	7/7	1/1	1/1	2/2	1/1	6/6	6/6
DL reproducibility checklist	7/7	6/6	3/3	6/6	1/1	6/6	2/2	3/2	2/2	7/7	5/5	3/3
PRIME 2.0	7/7	6/6	3/3	6/6	1/1	7/7	3/3	1/1	2/2	6/6	6/6	6/6
PRIME	7/7	6/6	3/3	6/6	1/1	7/7	2/2	1/1	2/2	2/5	6/5	6/6
Radiology AI cybersecurity checklist	7/6	6/5	3/3	4/4	1/1	6/7	1/1	1/1	2/2	1/1	6/6	6/6
CLEAR-E3	7/7	6/6	3/3	5/6	1/1	7/7	2/2	5/2	3/2	6/6	5/5	6/6
RIDGE	7/7	6/6	3/3	6/6	1/1	7/7	1/2	1/2	2/2	2/5	6/5	6/6
iCARE	7/7	6/6	3/3	6/6	1/1	6/7	1/2	1/2	2/2	2/5	6/6	6/6
CLAIM 2024 Update	7/7	6/6	3/3	7/6	1/1	7/7	1/2	1/2	2/2	6/7	6/5	6/6
ESCR position statement	7/7	6/6	3/3	5/6	1/1	7/7	6/3	3/2	3/2	2/5	6/5	6/6
Pediatric radiology AI guide	7/6	6/5	4/4	4/5	1/1	7/7	1/1	2/1	3/2	2/1	5/5	6/6
CAR AI Framework	7/7	6/6	3/3	5/6	1/1	7/7	1/2	1/1	2/2	2/5	6/5	5/6
Introductory guide to AI in IR	7/6	6/5	3/3	5/4	1/1	7/7	1/1	1/1	2/2	2/1	6/6	5/6

Values are presented as R1/R2, where the first value represents the rating assigned by reviewer 1 and the second value represents the rating assigned by reviewer 2. I1–I23 correspond to the 23 items of the original AGREE II instrument. Ratings range from 1 (strongly disagree) to 7 (strongly agree).

Table S1B. Item-level AGREE II ratings for the included guidance publications (items 13–23).

Guidance document	I13	I14	I15	I16	I17	I18	I19	I20	I21	I22	I23
PI-RADS AI Model Checklist	2/2	2/2	6/6	5/5	7/7	5/5	7/7	3/3	6/6	2/2	4/4
AOSR position statements	4/4	1/1	5/5	6/6	7/7	6/6	5/5	4/4	6/6	7/7	4/4
CLEAR	4/4	6/6	6/6	4/4	7/7	6/6	7/7	2/2	6/6	7/7	6/6
R-AI-DIOLOGY	1/1	1/1	6/6	6/6	7/7	6/6	7/7	4/4	5/5	7/7	4/4
DL reproducibility checklist	1/1	1/1	6/6	4/4	7/7	5/5	7/7	2/2	5/5	7/7	4/4
PRIME 2.0	1/1	1/1	6/6	6/6	7/7	6/6	7/7	6/6	6/6	3/3	4/4
PRIME	3/1	2/2	6/6	6/5	7/7	5/5	7/7	4/3	5/5	4/7	4/4
Radiology AI cybersecurity checklist	1/1	1/1	6/6	6/6	7/7	6/6	7/7	4/4	6/6	1/7	4/4
CLEAR-E3	1/4	2/6	6/6	5/5	7/7	6/6	7/7	2/2	6/6	6/7	5/5
RIDGE	1/1	1/1	6/6	6/6	7/7	5/6	7/7	4/3	6/6	4/7	4/5
iCARE	5/4	1/1	6/6	6/5	7/7	6/6	7/7	5/3	6/6	7/7	4/4
CLAIM 2024 Update	2/4	1/6	7/6	6/4	7/7	6/6	7/7	3/2	6/6	7/7	4/5
ESCR position statement	4/4	1/1	6/6	6/6	7/7	6/6	7/7	6/4	6/5	6/7	4/7
Pediatric radiology AI guide	1/1	1/1	6/6	6/5	7/6	6/5	7/6	3/2	6/5	7/7	4/7
CAR AI Framework	1/4	1/1	6/6	6/6	7/7	6/6	7/7	6/6	6/6	3/7	4/7
Introductory guide to AI in IR	1/1	1/1	5/6	6/6	7/7	6/6	7/7	6/4	5/6	7/7	4/5

Values are presented as R1/R2, where the first value represents the rating assigned by reviewer 1 and the second value represents the rating assigned by reviewer 2. I1–I23 correspond to the 23 items of the original AGREE II instrument. Ratings range from 1 (strongly disagree) to 7 (strongly agree).

Table S2. Overall quality ratings and recommendations before and after consensus.

Guidance document	Overall R1	Overall R2	Final quality	Recommendation R1	Recommendation R2	Final recommendation
PI-RADS AI Model Checklist	5	6	5	Yes, with modifications	Yes, with modifications	Yes, with modifications
AOSR position statements	4	5	5	Yes, with modifications	Yes, with modifications	Yes, with modifications
CLEAR	6	6	6	Yes	Yes	Yes
R-AI-DIOLOGY	4	5	5	Yes, with modifications	Yes, with modifications	Yes, with modifications
DL reproducibility checklist	5	5	5	Yes, with modifications	Yes, with modifications	Yes, with modifications
PRIME 2.0	5	5	5	Yes, with modifications	Yes, with modifications	Yes, with modifications
PRIME	4	5	5	Yes, with modifications	Yes, with modifications	Yes, with modifications
Radiology AI cybersecurity checklist	4	5	4	Yes, with modifications	Yes, with modifications	Yes, with modifications
CLEAR-E3	6	6	6	Yes	Yes	Yes
RIDGE	4	6	5	Yes, with modifications	Yes	Yes, with modifications
iCARE	5	6	5	Yes, with modifications	Yes	Yes, with modifications
CLAIM 2024 Update	6	6	6	Yes	Yes	Yes
ESCR position statement	5	6	6	Yes, with modifications	Yes	Yes
Pediatric radiology AI guide	5	5	5	Yes, with modifications	Yes, with modifications	Yes, with modifications
CAR AI Framework	5	6	5	Yes, with modifications	Yes	Yes, with modifications
Introductory guide to AI in IR	5	5	5	Yes, with modifications	Yes, with modifications	Yes, with modifications

Final overall quality was rated on a 1–7 scale and was not calculated as the mean of the six domain scores. Final overall quality ratings and recommendations were agreed by consensus. Recommendations were classified as Yes, Yes, with modifications, or No.

Table S3. Inter-rater reliability for AGREE II domain assessments.

AGREE II domain	ICC(2,1)	95%
D1 - Scope and Purpose	0.78	0.47-0.92
D2 - Stakeholder Involvement	0.78	0.48-0.91
D3 - Rigour of Development	0.78	0.49-0.92
D4 - Clarity of Presentation	0.31	-0.16-0.68
D5 - Applicability	0.72	0.32-0.90
D6 - Editorial Independence	0.36	-0.08-0.70

ICC - intraclass correlation coefficient; CI - confidence interval.

ICC values were calculated separately for each AGREE II domain using a two-way random-effects, absolute-agreement, single-measure model [ICC(2,1)] with 95% confidence intervals, based on the original independent ratings of R1 and R2 across all 16 included documents.
