# Supplementary material for: Artificial Intelligence Reporting Guidelines in Radiology: A Systematic Review
Source: Diagnostics (Basel). 2026 Sep 20;16(18):3054. doi: 10.3390/diagnostics16183054 (PMC13605318; doi:10.3390/diagnostics16183054)
Supplement: Supplementary file 1 [file diagnostics-16-03054-s001.zip › Supplementary File S5. Project-specific working interpretation guidance for the AGREE II assessment.pdf]

Supplementary File S5. Project-specific working interpretation guidance for the AGREE II assessment

The original AGREE II instrument, including its 23 items, six domains, 7-point rating scale, and overall assessment, was used without modification. Project-specific working interpretation guidance was developed to standardize the application of individual AGREE II items to reporting guidelines, checklists, position statements, and evaluation frameworks concerning AI in medical imaging. The guidance was piloted on three guidance documents before formal assessment to calibrate its application between reviewers and resolve interpretive ambiguities. The final working interpretations used during the formal assessment are presented below.

AGREE II item*	Domain*	Project-specific working interpretation
1	D1 - Scope and Purpose	Assessment focused on whether the document clearly defined its objective, intended outcome, and scope of application.
2	D1 - Scope and Purpose	For reporting checklists and related documents, assessment focused on whether the problems, tasks, or stages of AI research addressed by the recommendations were clearly specified.
3	D1 - Scope and Purpose	The target population was interpreted broadly to include the relevant clinical population, imaging modality, study type, intended use case, and other stated limitations of scope.
4	D2 - Stakeholder Involvement	Assessment considered the involvement of relevant professional groups, including radiologists, other clinicians, methodologists, statisticians, AI or informatics experts, regulators, and other appropriate experts.
5	D2 - Stakeholder Involvement	Assessment considered whether patients, members of the public, or other intended users had been involved or formally consulted. Participation of professional experts alone was not considered sufficient to fulfill this criterion.
6	D2 - Stakeholder Involvement	Target users could include, depending on the document, authors, reviewers, radiologists, developers, IT teams, hospital decision-makers, or regulators, and were expected to be clearly defined.
7	D3 - Rigour of Development	Assessment focused on whether the document described a systematic evidence-search process, including elements such as search strategies, databases, date ranges, search terms, and methods of evidence identification.
8	D3 - Rigour of Development	Assessment focused on whether explicit inclusion and/or exclusion criteria were provided for the evidence or

		sources used to develop the recommendations.
9	D3 - Rigour of Development	A general limitations section was not considered sufficient. Assessment focused on whether the quality, consistency, limitations, or gaps of the supporting body of evidence were explicitly evaluated.
10	D3 - Rigour of Development	Assessment focused on whether the methods used to formulate recommendations were described, such as Delphi procedures, consensus methods, voting, predefined agreement thresholds, or procedures for resolving disagreements.
11	D3 - Rigour of Development	In the context of AI, relevant benefits and risks included patient safety, bias, model errors or failure, privacy, cybersecurity, and risks related to clinical implementation.
12	D3 - Rigour of Development	Assessment focused on whether individual recommendations were explicitly linked to supporting evidence, for example through citations or evidence tables associated with specific recommendations.
13	D3 - Rigour of Development	Routine journal peer review alone was not considered equivalent to formal external review of the guidance document unless an external expert review process was explicitly described.
14	D3 - Rigour of Development	Assessment considered whether an updating procedure was specified, including elements such as the timing or triggers for updating, the responsible group, and the proposed updating process.
15	D4 - Clarity of Presentation	Assessment focused on whether the intended user could clearly determine what should be reported, evaluated, or implemented on the basis of the recommendations.
16	D4 - Clarity of Presentation	For non-clinical guidance documents, this item was interpreted as referring to clearly presented alternative approaches, implementation pathways, or assessment options relevant to the document's intended purpose.
17	D4 - Clarity of Presentation	Key recommendations were considered readily identifiable when they were clearly distinguished, for example through a dedicated checklist, table, summary, algorithm, or typographic emphasis.
18	D5 - Applicability	Assessment considered organizational, technical, legal, ethical, data-related, and competency-related facilitators and barriers to implementation.
19	D5 - Applicability	Practical implementation support could include forms, downloadable checklists, examples, templates, algorithms, or other explicit instructions for applying the recommendations.

20	D5 - Applicability	Resource implications were interpreted to include time, staffing, infrastructure, costs, training requirements, licensing, and computational burden.
21	D5 - Applicability	Monitoring or auditing criteria could include adherence indicators, post-deployment monitoring, model drift, safety surveillance, and the frequency of subsequent review or reassessment.
22	D6 - Editorial Independence	Assessment focused on whether funding sources were reported and the role of the funding body in development of the document was explicitly described. An explicit statement that no funding was received was also considered informative.
23	D6 - Editorial Independence	Disclosure of competing interests alone was not necessarily considered sufficient to fully address this item; assessment also considered whether procedures for managing identified conflicts of interest were described.

*AGREE II item numbers and domain names correspond to the original AGREE II instrument.

Brouwers MC, et al. AGREE II: Advancing guideline development, reporting and evaluation in health care. *CMAJ*. 2010;182:E839–E842.
